# A case report and a literature review of Myeloid/Lymphoid Neoplasm with Eosinophilia and PCM1::JAK2 rearrangement representing as B-cell acute lymphoblastic leukemia B-ALL

**DOI:** 10.1007/s00277-026-06757-z

**Published:** 2026-01-14

**Authors:** Luka Čemažar, Klara Šlajpah, Njetočka Gredelj Šimec, Helena Podgornik

**Affiliations:** 1 Department of Hematology, University Medical Centre Ljubljana, Ljubljana, Slovenia; 2 Faculty of Medicine, University of Ljubljana, Ljubljana, Slovenia; 3 Faculty of Pharmacy, University of Ljubljana, Ljubljana, Slovenia

**Keywords:** Eosinophilia, MLN-eo-TK, PCM1:JAK2, B-ALL

## Abstract

Myeloid/Lymphoid Neoplasms with Eosinophilia and Tyrosine Kinase Gene Fusions (MLN-eo-TK) represent a distinct and heterogeneous group of hematologic malignancies characterized by recurrent gene fusions involving tyrosine kinases, such as *PDGFRA*,* PDGFRB*,* FGFR1*,* JAK2*,* FLT3*,* ETV::ABL1* and other partner genes/variants. Among these, gene rearrangements involving *PCM1::JAK2* are rare and may present diagnostic challenges, particularly when manifesting as acute lymphoblastic leukemia (ALL). We describe a case of a patient who presented with B-cell acute lymphoblastic leukemia (B-ALL) with a *JAK2* rearrangement. After the induction therapy, strong myeloid proliferation in bone marrow without evidence of residual lymphoblasts was observed and *JAK2* rearrangement was recognized to be a consequence of translocation t(8;9)(p22;p24)] resulting in *PCM1::JAK2* fusion. This finding indicated the presence of an underlying chronic myeloid/lymphoid neoplasm, meeting criteria for MLN-eo-TK. Following an inadequate response to standard chemotherapy, salvage regimens incorporating targeted agents (JAK2 and BCL-2 inhibitors) and allogeneic bone marrow transplantation were administered, all of which unfortunately resulted in short-lived clinical benefit. The case highlights the importance of distinguishing de novo lymphoid malignancies from MLN-eo-TK, especially when *JAK2* rearrangements are detected. Recognition of the clonal myeloid component during or after lymphoid-directed therapy has important diagnostic and therapeutic implications, supporting the use of targeted JAK2 inhibition in addition to standard chemotherapy.

## Introduction

Recent advances in research, insights into disease biology, and developments in molecular genetics have prompted revisions to the classification of certain hematologic malignancies. Both the World Health Organization (WHO) and the International Consensus Classification (ICC) have updated the former category of “Myeloid/Lymphoid Neoplasms Associated with Eosinophilia and Rearrangement of *PDGFRA*,* PDGFRB*,* FGFR1*, or *PCM1::JAK2*”. This group is now classified as Myeloid/Lymphoid Neoplasms with Eosinophilia and Tyrosine Kinase Gene Fusions (MLN-eo-TK). The updated MLN-eo-TK category includes not only previously mentioned gene rearrangements, but also newly recognized fusions genes involving *FLT3*,* ABL1* gene (e.g., *ETV6::ABL1*), and other rare tyrosine kinase gene variants [1–3]. Although the term *eosinophilia* is present in the category´s name, clinical findings from a registry-based analysis of 135 patients with MLN-eo-TK, revealed hypereosinophilia in only 60% of evaluable patients [4]. The frequency of eosinophilia varied significantly depending on the specific fusion gene involved: it was present in 91% of patients with *FIP1L1::PDGFRA* and 100% with *ETV6::ABL1*, but only in 43% of those with *PDGFRB*, *FGFR1*, or *JAK2* fusion genes [4, 5].

The PCM1::JAK2 fusion gene arises from a chromosomal translocation t(8;9)(p22;p24) and has two other genetic variants, namely *ETV6::JAK2* (t(9;12)(p24.1;p13.2)) and *BCR::JAK2* (t(9;22)(p24.1;q11.2)) [6]. These fusions lead to a constitutive activation of JAK2-TK, a key driver in the pathogenesis of various hematological malignancies. JAK2-rearranged neoplasms exhibit a diverse and heterogeneous clinical presentation [7, 8]. In addition to MLN-eo-TK, *JAK2* rearrangements can also be found in *BCR::ABL1*-negative myeloproliferative neoplasms (MPN), myelodysplastic neoplasms (MDS), MDS/MPN overlap syndromes, B/T lymphomas, acute myeloid (AML), lymphoblastic (ALL) or mixed- phenotype acute leukemia (MPAL) [9, 10].

Compared to neoplasms with *PDGFRA* or *PDGFRB* rearrangements, MLN-eo-TK with *JAK2* rearrangements have a higher rate of progression and are associated with a poorer prognosis. Additional clinical characteristics associated with *JAK2* rearrangements include a strong male predominance with a reported male to female ratio of approximately 10:1. Monocytosis is observed in approximately 25% of cases. Secondary blast phase (BP) is the initial presentation in 10–30% of cases, while primary BP is rare and occurs in less than 5% of cases [4, 11].

This case report presents a rare clinical manifestation of a myeloid/lymphoid neoplasm with eosinophilia (MLN-eo) with *PCM1::JAK2* rearrangement that initially presented as B-cell acute lymphoblastic leukaemia (B-ALL). We describe the diagnostic challenges, the integration of targeted therapy alongside intensive chemotherapy, and the patient’s clinical course during follow-up.

## Case report

In January 2025, a 37-year-old male with no prior medical history or known family history of hematologic disorders was admitted to the Department of Hematology at the University Medical Centre Ljubljana. He presented with a 4-day history of abdominal pain localized below the left costal margin, accompanied by anorexia, profuse night sweats, and intermittent low-grade fever (maximum temperature 37.5 °C). Initial laboratory evaluation revealed marked leukocytosis, with a white blood cell (WBC) count of 83.6 × 10⁹/L, anemia (hemoglobin 92 g/L), and thrombocytopenia (platelet count 20 × 10⁹/L). Differential blood count showed a left shift, including blasts 33%, myelocytes 6%, metamyelocytes 4%, promyelocytes 3%, and band neutrophils 7%, with segmented neutrophils comprising 36% of leukocytes. Notably, eosinophilia (2.51 × 10⁹/L; 3%) and monocytosis (1.67 × 10⁹/L; 2%) were present, while basophils were absent.

**Fig. 1:**
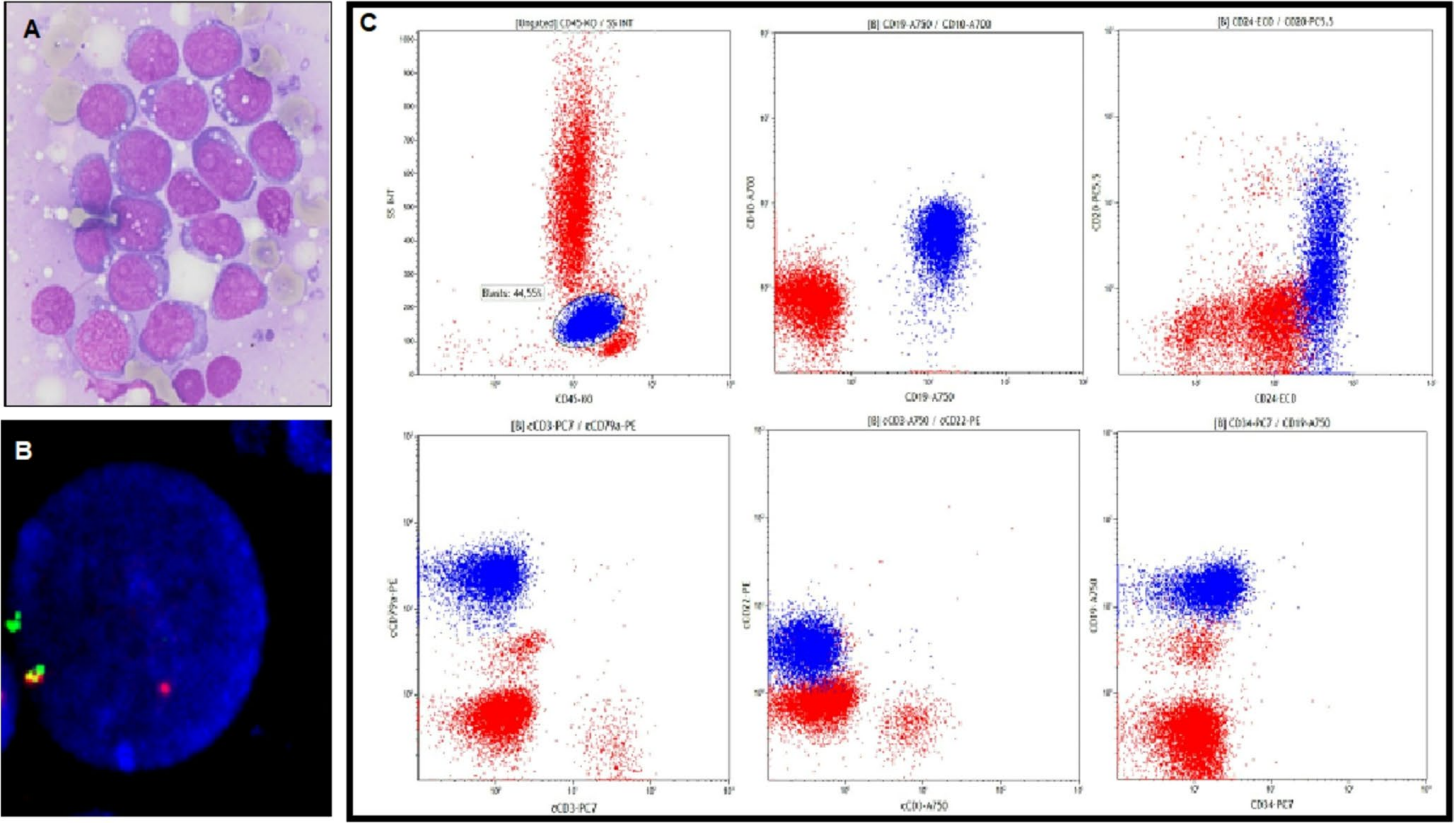
Diagnostic examination at the presentation of ALL. (**A**) Romanowsky staining of the bone marrow blasts. (**B**) JAK2 rearrangement, confirmed by interphase FISH analysis with the DNA probe JAK2 BA (Kreatech), visible as splitting of the fused yellow signal into a separate red and green signal. (**C**) The flow cytometric analysis shows the immunophenotype of B-ALL at diagnosis

Bone marrow aspiration and biopsy demonstrated extensive infiltration with blasts exceeding 90% of marrow cellularity. Morphologically, the blast cells exhibited basophilic cytoplasm with vacuolization (Fig. 1A), prompting further immunophenotypic characterization via flow cytometry. Immunophenotyping of the peripheral blood identified a clonal population comprising 45% of cells, with the following immunophenotypic profile: CD10⁺, CD19⁺, CD20⁺ (dim), CD22⁺ (dim), CD24⁺, CD38⁺, CD45⁺, CD58⁺, cytoplasmic CD22⁺, cytoplasmic CD79a⁺, HLA-DR⁺, and TSLP-negative (Fig. 1C). This immunophenotype was consistent with a diagnosis of B- common cell acute lymphoblastic leukemia (B-ALL).

Due to the dry tap, the availability of samples for cytogenetic and molecular analyses was limited. Fluorescence in situ hybridisation (FISH) detected a rearranged JAK2 gene on the short arm of chromosome 9 in 92% (184/200) of the nuclei examined, indicating a significant clonal population (Fig. 1B). Rearrangements involving *CRLF2*,* KMT2A*,* ABL2*,* MYC*,* PDGFRB*,* BCL6* and *BCL2* were excluded as well as a translocation t(9;22). Due to the poor chromosomal morphology and low mitotic index, the partner gene of *JAK2* was not recognized by conventional karyotyping, and NGS analysis of the RNA to detect the fusion was also not possible due to a lack of suitable samples. However, the presence of rearranged *JAK2* classified the disease as B-ALL with *BCR::ABL1*-like features.

For disease staging purposes, a CT and PET-CT scan using FDG labeling was performed. The scan revealed pathological bone marrow infiltration, with focally increased metabolic activity at multiple sites throughout the visualized skeleton—findings consistent with a primary hematologic malignancy. Additionally, hepatosplenomegaly was noted, with diffusely increased pathological FDG uptake in the spleen. Due to acute respiratory insufficiency and laboratory evidence of spontaneous tumor lysis syndrome, supportive treatment was initiated, including rasburicase, aggressive hydration, and dexamethasone, in accordance with the pre-phase regimen of the ALL treatment protocol. Based on the diagnostic workup, induction therapy was initiated according to the UKALL-14 protocol, which represents the standard treatment for newly diagnosed ALL at our center. The patient received, daunorubicin, vincristine, rituximab, pegasparaginase and intrathecal methotrexate as part of the induction regimen. Cerebrospinal fluid (CSF) infiltration was evaluated on two separate occasions: at diagnosis and on day 14 of the induction cycle. Flow cytometry was negative for malignant hematopoietic cells. At the beginning, the patient also reported paresthesias in his chin and legs, so we performed an MRI of the entire spinal axis. The MRI did not reveal spinal cord infiltration. However, it did show a suspicious infiltration of the calvarial diploe and a more intense enhancement of the adjacent intracranial dura. Based on the spinal fluid results, we concluded that these findings were not sufficient to suggest leukemic infiltration. In the later stages, we did not re-examine the spinal fluid, nor did the patient experience any neurological deficits. At the end of induction, measurable residual disease (MRD) assessment was performed by flow cytometry, still detecting 0.004% of lymphoblasts, which was below the quantification threshold of 0.010% which is also the limit for MRD positivity. Given the presence of unfavorable cytogenetics and detectable MRD, the decision was made to proceed with allogeneic hematopoetic stem cell transplantation (allo-HCT), and a donor search was initiated.

**Fig. 2:**
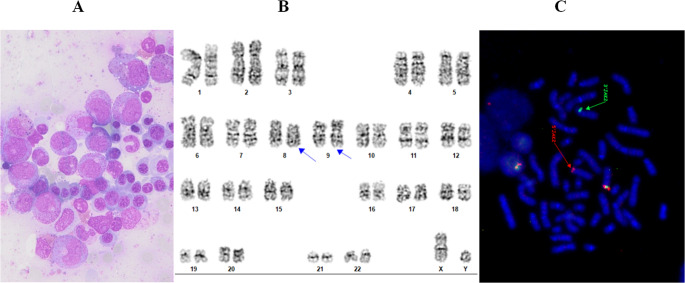
Diagnostic work up after induction chemotherapy. (**A**) Bone marrow cells with increased granulopoiesis and erythropoiesis Translocation t(8;9) observed by (**B**) chromosome banding analysis of cultivated peripheral blood cells, (**C**) as a rearrangement of *JAK2* gene on 9p (JAK2 BA probe Kreatech)

Before the planned admission for the second induction, on day 36 after the first induction, the patient was admitted due to marked leukocytosis and poor general condition. Laboratory testing revealed marked leukocytosis with a WBC count of 83.4 × 10⁹/L, although no blast cells were detected. The differential count showed elevated segmented neutrophils 50%, lymphocytes 9%, monocytes 2%, and immature granulocytes, including band neutrophils 6%, metamyelocytes 14%, myelocytes 11%, and promyelocytes 8%, no eosinophils were detected. Bone marrow was hypercellular with signs of regeneration from previous aplasia due to intensive chemotherapy, characterized by increased granulopoiesis and erythropoiesis (Fig. 2A). MRD in the bone marrow was positive notably time point (0.016%) of B-lymphoblasts was detected. The predominant population in the bone marrow was of myeloid lineage with maturation.

As the initial chromosome banding analysis was unsuccessful, the test was repeated using peripheral blood. A balanced translocation t(8;9)(p22;p24), was detected in all analyzed metaphases (Fig. 2B), and the *JAK2* gene was confirmed as rearranged (Fig. 2C) in 46% of interphase nuclei by FISH. Next generation sequencing (NGS) performed on peripheral blood confirmed the *PCM1::JAK2* fusion gene, consistent with the t(8;9)(p22;p24) translocation observed by karyotyping. The fusion gene was detected on isolated RNA using anchored multiplex PCR for library preparation. Selected gene regions were enriched with an amplicon-based approach using the FusionPlex ALL kit (ArcherDX, Colorado, USA). In addition to fusion genes, the dedicated panel can also detect mutations. However, no variants were detected in any of the targeted genes in the fusion panel or by NGS DNA analysis using the VariantPlex Core Myeloid panel (ArcherDX, Colorado, USA).

**Fig. 3:**
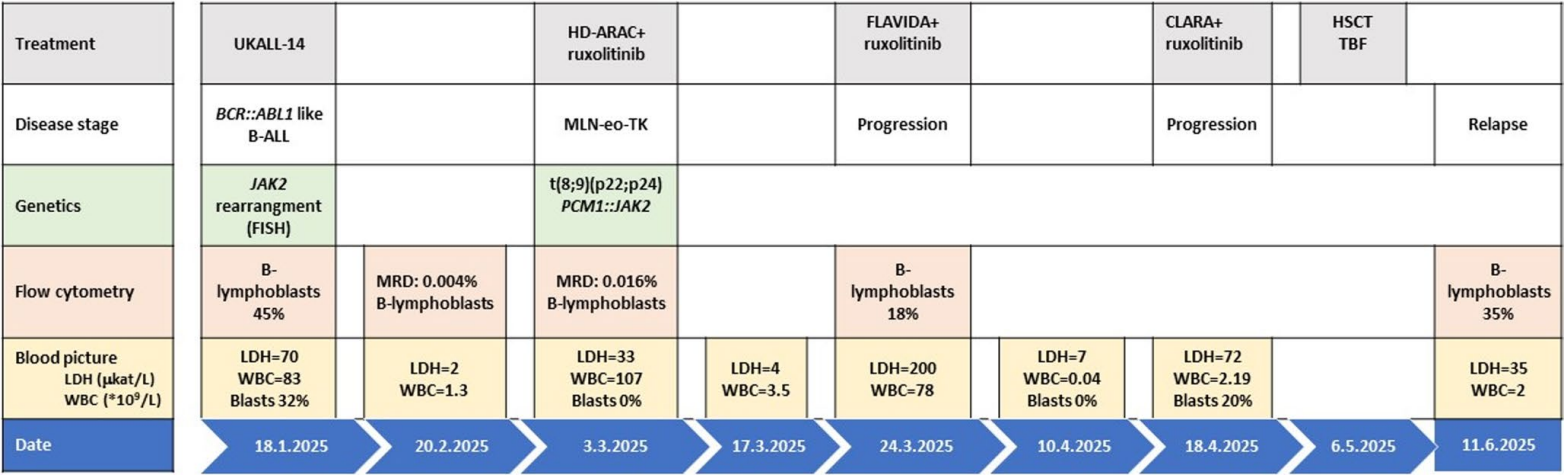
Timeline of disease stage, key diagnostic markers, and treatment. UKALL-14: induction therapy for adults with acute lymphoblastic leukemia; HD-ARA: high-dose cytarabine; FLAVIDA: fludarabine, cytarabine, idarubicin + venetoclax; CLARA: clofarabine, cytarabine; TBF: Thiotepa-busulfan-fludarabine; HSCT: hematopoietic stem cell transplantation

Due to disease progression and significant leukocytosis, cytoreduction was initiated immediately. The patient was started on high-dose cytarabine (HD Ara-C) at 3 g/m² combined with targeted therapy using ruxolitinib 20 mg twice daily (BID). However, due to treatment-related cytopenias, the ruxolitinib dose was reduced to 10 mg BID. The therapeutic effect was short-lived, with recurrent leukocytosis and elevated LDH (Fig. 3). Repeated flow cytometry analysis confirmed 18% B-lymphoblasts with an unchanged immunophenotype in peripheral blood. Given the primary resistance to prior lines of therapy, treatment was escalated to a FLAVIDA protocol (fludarabine 30 mg/m^2^ on days 2–6, cytarabine 1.5 g/m^2^ on days 2–6, idarubicin 6 mg/m^2^ on days 4–6, venetoclax 400 mg on days 1–7) while continuing ruxolitinib at a reduced dose (10 mg BID). Notably, after each intensive regimen, the patient developed a clear aplastic phase (11–17 days) before rapidly reverting to a proliferative phase, indicating transient chemosensitivity but failure to achieve durable disease control. On day 21, 20% blasts were detected in the peripheral blood. We decided to proceed with bridging therapy to transplantation using the CLARA protocol. As only a haploidentical donor was available, a sequential haploidentical HSCT was planned. As a bridging strategy prior to transplantation, the CLARA regimen (clofarabine 30 mg/m² administered intravenously on days 1–5, followed by intermediate-dose cytarabine 1 g/m² intravenously on days 1–5) combined with ruxolitinib was initiated. Because the primary objective was to proceed to sequential haploidentical HSCT as early as possible, no repeat bone marrow evaluation was performed to formally document remission status before transplantation. The patient experienced a transient aplastic phase after CLARA, indicating some chemosensitivity.

On May 6, 2025, myeloablative conditioning with the TBF regimen (thiotepa 5 mg/kg on days − 6 and − 5; fludarabine 30 mg/m^2^ on days − 4 to −2 and the total dose of i.v. busulfan 9 mg/kg days − 4 to −2) was started. The conditioning regimen was well tolerated, with no early complications. On May 12, 2025, the patient received 5.24 × 10⁶ CD34⁺ cells/kg from a haploidentical donor. The transplant procedure was uneventful. Graft-versus-host disease (GVHD) prophylaxis included cyclosporine and mycophenolate mofetil, with post-transplant cyclophosphamide administered at 50 mg/kg on days + 3 and + 5. All immunosuppressive treatments were well tolerated. Peri-transplant ruxolitinib therapy, started before conditioning, was maintained throughout the conditioning phase at a reduced dose and later tapered to a maintenance dose of 5 mg BID. During hospitalization, the patient developed CMV viremia and BK virus–associated hemorrhagic cystitis, both of which were managed appropriately. Chimerism analysis on day + 28 revealed mixed chimerism with 4% recipient cells. On day + 30 the presence of circulating blasts and elevated LDH levels indicated early relapse, which was confirmed latter by flow cytometry showing 35% of B-lymphoblasts.

Given the aggressive and refractory disease course, palliative care was initiated. The patient sadly passed away two days later.

## Discussion

In the context of this case report, the main challenge was to distinguish between de novo hematopoietic neoplasms and those arising by transformation from a pre-existing myeloid or lymphoid disorder. The initial presentation of MLN-eo-TK in the blast phase as B-ALL is rare, while its recognition in a chronic phase underpins the stem cell origin of the disease and helps to distinguish it from de novo lymphoid malignancies [3, 7, 12].

The 2023 ICC of Eosinophilic Disorders offers updated and more precise guidance for distinguishing MLN-eo-TK (especially those presenting as ALL) from *BCR::ABL1*-like B-ALL and de novo T-ALL [3]. In particular, *JAK2* fusion partners have been identified alongside *PCM1* — including *BCR* and *ETV6*— in both B- and T-lineage leukemias, further complicating diagnostic classification and therapeutic decision-making [13]. However, *PCM1::JAK2* fusion is rarely observed in other lymphoproliferative malignancies (such as T-cell lymphomas) [9, 14]. When MLN-eo-TK presents as ALL (either B-ALL or T-ALL)—there should be involvement in both lymphoblasts and cells of myeloid lineage. If an underlying chronic myeloid neoplasm is present — either prior to, during, or after treatment of the lymphoid component (in our case B-ALL) - this supports a more accurate classification as MLN-eo-TK. On the other hand, *BCR::ABL1*-like B-ALL may have a *JAK2* rearrangement with other partner genes (*STRN3::JAK2* or *PAX5::JAK2*), which by definition are not MLN-eo-TK [15].

At presentation, our patient had peripheral eosinophilia (2.51 × 10⁹/L; 3%) and concurrent monocytosis (1.67 × 10⁹/L; 2%), which was only a consequence of the highly elevated WBC and therefore at first did not indicate underlying MLN-eo-TK. That being said, according to established criteria, the eosinophil count exceeded 1.5 × 10⁹/L, meeting the definition of hypereosinophilia (HE). Based on severity grading, this value corresponds to moderate eosinophilia (AEC 1.5–5 × 10⁹/L), whereas severe eosinophilia is defined as an AEC > 5 × 10⁹/L [16, 17].

This is consistent with previous reports that hypereosinophilia is less common in *JAK2* fusions [4]. The extensive infiltration of the bone marrow by leukaemic blasts (> 90%) made it impossible to assess underlying dysplastic or proliferative changes in granulopoiesis and erythropoiesis, as well as alterations in megakaryocytes, bone marrow fibrosis or mast cell proliferation, which are frequently reported in similar contexts [5]. Initial cytogenetic analysis was limited by low mitotic index and poor chromosomal morphology, which precluded identification of the t(8;9)(p22;p24) translocation involving *JAK2*. Consequently, the *JAK2* rearrangement was initially interpreted within the diagnostic and prognostic framework of ALL, i.e. the diagnosis was B-ALL with a *BCR::ABL1*-like gene expression profile based on the *JAK2* rearrangement.

In this case, the distinction became clearer when the patient entered the regeneration phase following aplasia induced by intensive chemotherapy. Repeated bone marrow aspirations subsequently demonstrated a detectable MRD (0.016%) with the same immunophenotype of lymphoblasts as at initial diagnosis, while blood parameters and the bone marrow itself showed marked hypercellularity and proliferation resembling a myeloproliferative neoplasm (Fig. 2A). Cytogenetic analysis confirming t(8;9)(p22;p24) and RNA sequencing detecting a *PCM1::JAK2* fusion were crucial to revise diagnosis to MLN-eo-TK with *PCM1::JAK2* presenting as B-ALL in the blast phase. In all subsequent analyses, the B-lymphoblast population with an unchanged immunophenotype was consistently detected by flow cytometry. Our case highlighted the importance of a second evaluation when chromosome banding analysis fails at diagnosis. Especially in acute leukemias with suggestive molecular features, a second evaluation after disease burden has been reduced can be highly informative.

In MLN-eo-TK involving *PCM1::JAK2* treatment response and prognosis, particularly progression-free survival (PFS) and overall survival (OS), are closely tied to the disease phase at diagnosis. Patients who present in the chronic phase (e.g., MPN or MDS/MPN overlap) tend to have significantly better outcomes than those diagnosed with acute leukemia or in blast phase (BP) [12].The overall survival rate is approximately 60% for chronic-phase disease, compared to 15% for AML. The median OS from onset of blast phase in *PCM1::JAK2* cases is 1.7 years (range: 0.1–5.5 years). In a systematic review by Kaplan et al., the 5-year survival rate for patients with *PCM1-JAK2* fusion-associated ALL was reported to be 40.0% (6.6–73.4%) [18].

Following NCCN recommendations, management of MLN-eo-TK depends on disease extent (BM/PB blasts ≥ 20%), the presence of extramedullary disease, and lineage involvement. Tyrosine kinase inhibitors (TKIs) are available for all major fusion genes, but early consideration of allo-HSCT is recommended in eligible patients, particularly for *JAK2*,* FGFR1*,* FLT3*, or *ETV6::ABL1* fusions [10]. In our case, the patient was an allo-HSCT candidate from the outset (initial diagnosis: Ph-like B-ALL) and remained a candidate after revision to MLN-eo-TK with *PCM1::JAK2* rearrangement. Consistent with current practice, ruxolitinib was not incorporated into frontline induction for Ph-like ALL; outside clinical trials, NCCN emphasizes ABL-class TKIs (e.g., dasatinib) when an ABL-class lesion is present, whereas JAK-pathway inhibition is investigational (e.g., ruxolitinib in COG AALL1521 and other early-phase studies) [19–21]. According to previous studies, we added ruxolitinib at the time of salvage/bridging therapy once the diagnosis was revised to MLN-eo-TK with *PCM1::JAK2*, and proceeded toward allo-HSCT. This approach aligns with recent expert guidance and registry data indicating that, for JAK2-fusion to MLN-eo-TK, TKIs (e.g., ruxolitinib) can be used to bridge to transplant, while allo-HSCT remains the only potentially curative option in eligible patients [19]. In our patient, ruxolitinib produced virtually no clinical or hematologic benefit, consistent with the marked heterogeneity in JAK-inhibitor sensitivity reported in *PCM1::JAK2*-rearranged neoplasms. We therefore continued with CLARA bridging therapy to allo-HSCT as clofarabine-containing regimens showed meaningful activity in relapsed/refractory AML with efficacy demonstrated towards both cell lines in vitro and is widely used as bridge-to-transplant strategies, as demonstrated in previous studies [22–25]. In our patient, CLARA combined with ruxolitinib was likewise employed as bridging therapy in the context of rapidly progressive, heavily pretreated disease and limited alternatives.

To further expand on the targeted therapy approach, we reference the study by Schwaab et al. (2015) which examined the efficacy of ruxolitinib, in treating myeloid neoplasms with *PCM1::JAK2* and *BCR::JAK2* fusion genes. The research highlights that while ruxolitinib can induce a complete clinical, hematologic, and cytogenetic response in patients, the duration of remission is limited. In the study, two male patients with these fusion genes achieved complete remission after 12 months of ruxolitinib treatment. However, the remission was short-lived, with relapses occurring after 18 and 24 months, respectively. The study underscores the importance of cytogenetic analysis in guiding targeted therapy and suggests that ruxolitinib may serve as a bridging therapy before allo-HSCT [26, 27].

Regarding the ruxolitinib dosing a phase I/II trial by Goulart et al.l., examined the dosing in patients with relapsed and/or refractory Philadelphia-like ALL. Study enrolled 10 patients in the ruxolitinib cohort. No dose-limiting toxicities were observed in the two dosing cohorts (15 mg BID and 20 mg BID). However, the third cohort (25 mg BID) was terminated early due to slow accrual. The most common severe adverse events were related to infectious complications. Regarding efficacy, the trial observed low overall efficacy, with only 1 out of 10 patients in the ruxolitinib cohort achieving complete remission with incomplete platelet count recovery [20].

Several disease- and patient-specific factors may account for the lack of activity: (i) aggressive/blast-phase biology, in line with the B-ALL report by Wouters et al. where ruxolitinib reduced disease burden but did not replace intensive therapy; (ii) co-existing cytogenetic and molecular abnormalities, as described in modern MLN-eo-TK series, which may drive disease independently of *JAK–STAT* signaling; and (iii) possible lineage- or subclone-restricted involvement of the *PCM1::JAK2* fusion, such that a substantial fraction of blasts was not dependent on JAK2 signaling, resulting in an overall poor clinical response [28]. One additional reason for the ineffectiveness of ruxolitinib could be variants in *JAK2*, which have been linked to resistance [29]. However, this is less likely in our case, as *JAK2* mutations which were screened after induction therapy were found to be absent, and the duration of ruxolitinib treatment was too short for resistant variants to be acquired later.

In their 2024 systematic review of MLN-eo-TK, Lübke et al. emphasized that majority of cases benefit from the use of TKIs, and they highlighted the potential therapeutic role of fedratinib— a newer selective JAK2 inhibitor — particularly in cases involving *JAK2* rearrangements [30]. Updated guidelines recognize additional JAK2 inhibitors such as: fedratinib, momelotinib, pacritinib as a treatment options in the case of ruxolitinib unavailability or intolerability [5]. Contemporary reviews note that this agents could theoretically be considered in first- or second-line settings, or in transplant-ineligible patients, but clinical data in JAK2-rearranged MLN-eo-TK are lacking [5, 28]. In this context, we did consider, at least conceptually, whether a switch to fedratinib on a compassionate-use basis might be appropriate once ruxolitinib inefficacy became evident. Given that allo-HSCT remains the only potentially curative option for eligible *PCM1::JAK2* patients, we prioritized achieving adequate cytoreduction to proceed to transplantation rather than initiating a second, unvalidated JAK inhibitor.

The role of allo-HSCT in nonclassical MPNs and MDS/MPNs further addresses the recommendations from the Chronic Malignancies Working Party (CMWP) of the European Society for Blood and Marrow Transplantation (EBMT). The guidelines emphasize the importance of early identification of potential candidates for allo-HSCT which is particularly crucial for patients with chronic neutrophilic leukemia, chronic eosinophilic leukemia (CEL), and MLN-eo-TK. Although the data on JAK2 rearrangements were limited by the small number of transplants (according to EBMT only six patients in Europe with *JAK2* rearrangements received alloHSCT between 2016 and 2023), they still provided valuable insights [26]. Published series also highlight the rarity of such cases and the limited numbers of transplants, underscoring the need for early referral [5].

In the later stages of treatment, (-due to poor response to previous lines of therapy-), we decided to treat the patient based on the results of a retrospective analysis by Richard-Carpentier et al., in which venetoclax, a selective BCL2 inhibitor, was used in combination with chemotherapy for lymphoma (R/R T-ALL). The study was a retrospective review of 13 patients treated at the authors’ institution. The authors concluded that the combination of venetoclax and chemotherapy shows promising efficacy in treating R/R T-ALL, suggesting that further studies are needed to explore the clinical benefits of BCL2 inhibitors in this context. The study reported a median overall survival of 7.7 months and a relapse-free survival of 4.0 months [31]. To our knowledge, venetoclax use has not been reported in MLN-eo-TK with *PCM1::JAK2* fusions. Evidence for BCL2 inhibition in this setting is limited to anecdotal reports, including a recent case describing prolonged disease control with ruxolitinib plus venetoclax in a *BCR::JAK2*-rearranged myeloid neoplasm, suggesting potential synergistic activity of JAK2 and BCL2 blockade [32].

## Conclusion

Our case illustrates the diagnostic complexity and aggressive clinical course of MLN-eo-TK with *PCM1::JAK2* rearrangement, which initially presented as B-ALL. The evolving disease phenotype, limited availability of diagnostic material at presentation, and overlapping features with *BCR::ABL1*-like ALL delayed definitive classification. Reassessment during hematologic regeneration was crucial for accurate diagnosis. Despite the integration of targeted therapy and multiple lines of intensive chemotherapy, the disease demonstrated primary resistance and rapid progression, emphasizing the poor prognosis of *PCM1::JAK2*-rearranged MLN-eo-TK in BP. This case reinforces the importance of early genetic testing, and highlights the need for continued research into more effective therapeutic strategies for this rare and high-risk entity.

## Data Availability

No datasets were generated or analysed during the current study.

## Declarations

In accordance with the Declaration of Helsinki, written informed consent was obtained from the patient for the use of biological material and data obtained through analysis for research purposes, while maintaining anonymity. The patient also consented to the publication of the results for scientific purposes.
